# Multiview deep learning improves detection of major cardiac conditions from echocardiography

**DOI:** 10.1038/s44161-026-00786-7

**Published:** 2026-03-17

**Authors:** Joshua P. Barrios, Minhaj U. Ansari, Jeffrey E. Olgin, Sean Abreau, Jacques Delfrate, Elodie L. Langlais, Robert Avram, Geoffrey H. Tison

**Affiliations:** 1 Department of Medicine, Division of Cardiology, University of California, San Francisco, San Francisco, CA USA; 2 Bakar Computational Health Sciences Institute, University of California, San Francisco, San Francisco, CA USA; 3 Center for Biosignal Research, University of California, San Francisco, San Francisco, CA USA; 4 Division of Cardiology, Department of Medicine, Montreal Heart Institute, Montreal, Quebec Canada; 5 Department of Biomedical Engineering, École Polytechnique de Montréal, Montreal, Quebec Canada; 6 Department of Medicine, University of Montreal, Montreal, Quebec Canada

**Keywords:** Cardiology, Medical imaging, Machine learning

## Abstract

Medical imaging often captures multiple two-dimensional views of three-dimensional anatomic structures, but most artificial intelligence (AI) models analyze two-dimensional data. Here we show that integrating multiple imaging views using a single AI model can improve diagnostic performance. We developed a deep neural network (DNN) architecture that combines information from multiple video views simultaneously. Using echocardiogram data from the University of California, San Francisco, and the Montreal Heart Institute, we applied our multiview DNN approach for three primary demonstration tasks: detecting any left or right ventricular abnormality, diastolic dysfunction, and substantial valvular regurgitation. Across various tasks, our multiview DNNs improved discrimination as measured by the area under the receiver operating characteristic curve by 0.06–0.09 compared to DNNs trained on any single view. This demonstrates that AI models that can combine information from multiple imaging views simultaneously can better capture complex anatomy and physiology for certain tasks, underscoring the value of a multiview paradigm for AI in medical imaging.

## Abstract (web summary)

Barrios et al. develop a multiview deep neural network to jointly analyze multiple echocardiographic video views, improving detection of cardiac anatomy and function.

## Main

Medical imaging plays a critical role in cardiovascular medicine, providing insights into anatomic structure, hemodynamics, and function. Although cardiac anatomic structure is three-dimensional (3D), most imaging modalities capture multiple two-dimensional (2D) tomographic image slices of 3D anatomic structures. These multiple 2D slices, also called views, carry complementary information that physicians must interpret to reconstruct a 3D mental model, enabling optimal assessment of the structural or functional characteristics that distinguish disease from non-diseased states. Cardiac ultrasound, or echocardiography (echo), is an example of a common imaging modality that often captures >100 distinct views that together provide 3D information about the heart beating in time.

Advances in artificial intelligence (AI) methods^1–3^ have facilitated analysis of medical imaging, particularly computer vision which uses AI to analyze raw images and videos. The advent of deep neural networks (DNNs) provided the first major advance by enabling analysis of an image’s raw pixels, followed more recently by the development of DNNs that analyze raw video data by integrating information over time across the sequential images of a video^4–6^. Such DNNs trained using either image or video data have been successfully applied in medicine to detect various diseases^7–14^. However, these existing DNN architectures are poorly suited to integrate multiple 2D imaging views simultaneously as physicians do to accurately comprehend a 3D structure. In cardiac echo, for example, reliable diagnosis most often depends upon the corroboration of information contained across multiple echo views. An interpreting cardiologist routinely performs this mental integration of information as each view captures complementary information from a different perspective.

In this Article, we developed a purpose-built multiview DNN architecture that is specifically designed to integrate 3D video information from multiple complementary 2D imaging views mirroring how a physician interprets 3D anatomic structures. Our multiview DNN architecture simultaneously accepts inputs from multiple views while also being designed to integrate information between these views through dedicated DNN layers. Our multiview DNN architecture uses a mid-fusion approach to combine features from each input view at an intermediate stage enabling the network ample opportunity to integrate inter-view information. Whereas the primary innovation of image-based DNNs was integration of the 2D spatial information of raw image pixels, and the primary innovation of video-based DNNs was integration of temporal information across time (captured by the sequence of frames), our multiview DNN enables integration of spatiotemporal information from multiple views simultaneously to accomplish the target task.

Here we applied the multiview DNN architecture to three primary demonstration tasks in cardiac echo, a commonly obtained medical imaging modality. We show that our multiview DNN architecture readily outperforms standard video-based DNNs trained on any single view for each of the three tasks, which include both standard echo diagnoses and “novel” echo diagnoses—or diagnoses that cannot typically be made by cardiologists using echo data alone.

## Results

**Fig. 1:**
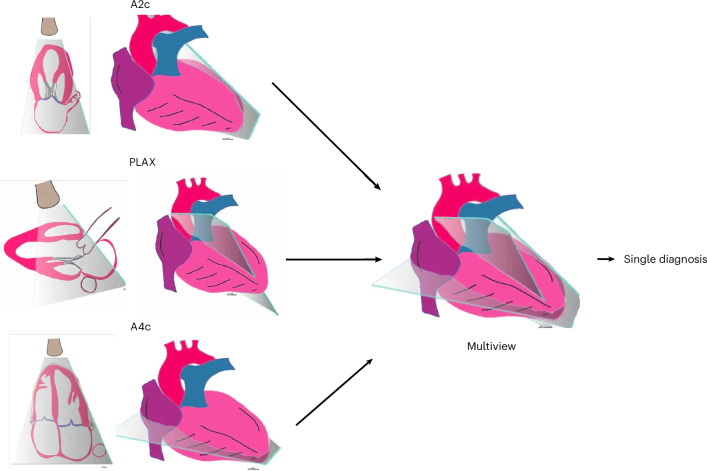
Integrating multiple 2D tomographic images of a 3D structure. Each single imaging view contains detailed 2D information about a slice (tomograph) of a 3D structure, such as the heart. Multiple 2D tomographic views often contain distinct and complementary information about different anatomic structures contained within that view. To obtain the most comprehensive assessment of any single diagnosis within a complex organ such as the heart, it is necessary to simultaneously consider information from multiple views at once. This is routinely performed by cardiologists who interpret echocardiograms of the heart. This practice of simultaneously considering differential information from multiple imaging views provides the conceptual basis for the development of the multiview DNN architecture. Images copyright Atif Qasim; reproduced with permission.

**Fig. 2:**
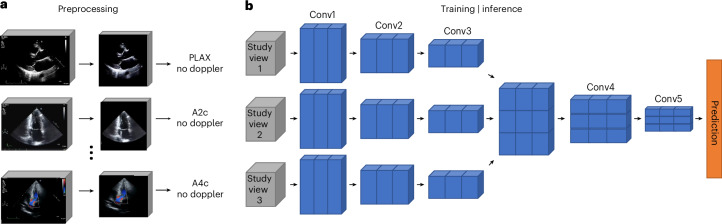
Multiview DNN and data processing pipeline. Multiview neural network architecture and data preprocessing for the demonstration imaging modality of cardiac echo. **a**, All videos from a single echo study undergo standardized preprocessing (masking to exclude nonmoving pixels and pixels outside the ultrasound region, cropping to ultrasound image region and resizing to 224 × 224 pixels). Each echo video’s view and presence of color-doppler signal are detected using trained view-classification and doppler-detection DNNs. **b**, One echo video from each predefined echo view (A4c, A2c, and PLAX) accepted by the multiview DNN is selected from the same echo study; these three echo videos are used as simultaneous inputs into the multiview DNN classifier to predict the target task. Embeddings from each video are passed through individual convolutional encoder blocks (Conv1–Conv3) before they are concatenated along a new dimension and then passed through two more convolutional blocks (Conv4 and Conv5) that perform cross-view convolutions to integrate spatiotemporal information between views before the final prediction is made.

**Extended Data Fig. 1:**
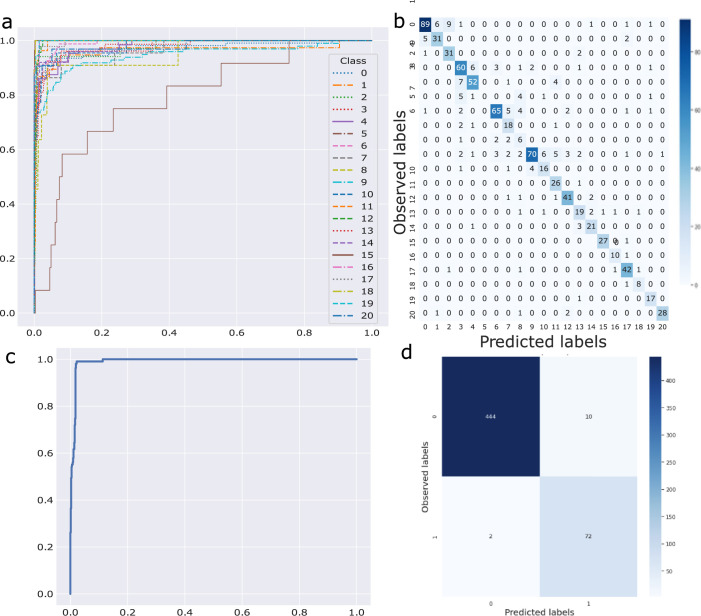
Performance of video-based echo 21-view classifier and color doppler detector. (**A**) Receiver operating characteristic curves and (**B**) confusion matrix for the 21 class echo view classifier. The view classifier DNN achieved a mean AUC of 0.972 across 21 classes. (**C**) Receiver operating characteristic curve and (**D**) confusion matrix for the DNN color doppler classifier. The color doppler classifier achieved an AUC of 0.991. View class number view labels: 0: parasternal long axis, 1: parasternal long axis (zoom on left atrium), 2: parasternal long axis (standard zoom), 3: apical two chamber, 4: apical two chamber (left atrium occluded), 5: apical two chamber (left ventricle occluded), 6: apical three chamber, 7: apical three chamber (left atrium occluded), 8: apical three chamber (left ventricle occluded), 9: apical four chamber, 10: apical four chamber (left ventricle occluded), 11: apical four chamber (left atrium occluded), 12: apical five chamber,13: parasternal short axis apical level 14: parasternal short axis (mitral valve level), 15: parasternal short axis (zoom on left atrium), 16: parasternal short axis (aortic valve zoom), 17: subcostal, 18: suprasternal, 19: right ventricle inflow, 20: other.

The multiview DNN architecture takes in multiple imaging views at the same time (Fig. 1) and achieves the task(s) it is trained for by learning patterns both within each raw video view and across the views together (Fig. 2). Cardiac echo diagnosis typically requires triangulating information simultaneously across multiple views making it an ideal demonstration modality for the multiview DNN. We trained each of the three demonstration multiview DNNs using three predefined echo views (the views most clinically suitable for each task) obtained from the same echo study for each patient. Training and internal validation echo data was derived from adult patients that received transthoracic echoes from 2012 to 2020 at the University of California, San Francisco (UCSF). All videos were masked and cropped to exclude any burned-in text and annotations and resized to 224 × 224 pixels. We first trained a video-based DNN view classifier to distinguish between 21 echo view classes and another video-based DNN doppler classifier to detect the presence of color doppler within the video. Performance metrics for these view/doppler DNN classifiers are provided in the supplement (Extended Data Fig. 1). We then applied the view and doppler DNN classifiers to all echos in the UCSF dataset to identify the specific echo videos to train and validate each of the three demonstration tasks.

Our multiview DNN architecture uses 3D convolutions first to integrate spatial and temporal information across multiple frames of a single video^15^. The multiview DNN architecture is designed around a video-based DNN backbone—here we used the “Expand 3D”, or “X3D”^15^ video-based DNN—which allows for other DNN backbones to be used as suitable for future applications. The core innovation of our multiview DNN architecture is the use of dedicated convolutional neural network layers that integrate information between all input views to accomplish the target task (Fig. 2). This allows the DNN to learn patterns within each input video and also the patterns between multiple videos, such as the motion of a heart valve captured over time from multiple views. Additional technical details are described in Methods.

To examine the performance of our multiview DNN architecture, we trained separate single-view DNNs representing the current state of the art^7,16–18^ for each individual view for our three echo tasks. We selected two “standard” composite echo tasks where accurate manual interpretation requires corroboration from more than one view: identification of left or right ventricular (LV/RV) abnormalities and identification of substantial valvular regurgitation (moderate severity or greater in the tricuspid, mitral, or aortic valves). We also selected one “novel” echo task that cannot typically be interpreted via physician manual interpretation using non-doppler, brightness mode (B-mode) echo: identifying diastolic dysfunction. There is no standard approach for cardiologists to manually interpret diastolic dysfunction using non-doppler B-mode echo videos alone, making this a novel AI-enabled echo task.

To derive reference-standard labels for training and testing for each task, we obtained assessments from clinical echo reports that were interpreted by level-3 echo board-certified cardiologists in the UCSF echo lab. Ventricular abnormality was defined as positive if there was any abnormality in LV/RV size or function^19^. Diastolic dysfunction was defined as any diastolic dysfunction (grades 1–4) as determined by American Society of Echocardiography (ASE) guidelines^20,21^. Substantial valve regurgitation was defined as moderate or greater regurgitation in any of the mitral, tricuspid, or aortic valves according to ASE guidelines^22^. For the ventricular abnormality and diastolic dysfunction multiview DNNs, the three input echo views used were non-doppler apical four-chamber (A4c), apical two-chamber (A2c), and parasternal long-axis (PLAX) views; for valve regurgitation, the input echo views were color-doppler A4c, apical five-chamber (A5c), and PLAX views.

### Multiview and single-view performance on composite endpoints

To train the LV/RV abnormality multiview DNN, we identified a cohort of 41,790 echo studies from 20,504 patients at UCSF that had all LV and RV measurements available in the clinical echo report. The cohort had a mean age of 63 years (standard deviation (s.d.) 17 years), and 50.3% were female (Table 1). The prevalence of any LV/RV abnormality in this cohort was 24.5%. Data were split by patients into training, development, and testing datasets. Hyperparameters and model checkpoints were chosen based on the development dataset, and final performance metrics were calculated on the held-out test dataset. Additional details are provided in Methods.

**Table 1:** UCSF echo characteristics by cohort

Characteristic	Ventricular abnormality	Diastolic dysfunction	Valve regurgitation
Patients	20,504	6,643	18,573
Age, mean (s.d.)	63 (17)	65 (16)	61 (17)
Female^a^	10,321 (50.3%)	3,345 (50.3%)	9,300 (50.0%)
Male^a^	10,148 (49.5%)	3,290 (49.5%)	9,248 (49.8%)
Race			
White	10,776 (52.6%)	3,482 (52.4%)	9,978 (53.7%)
Asian	3,577 (17.4%)	1,214 (18.3%)	3,171 (17.1%)
Latinx	2,208 (10.8%)	775 (11.7%)	2,007 (10.8%)
Black	1,744 (8.5%)	531 (8.0%)	1,542 (8.3%)
Other	2,092 (10.2%)	634 (9.5%)	581 (3.1%)
Echo studies	41,790	11,411	27,692
Hypertension	26,909(64.4%)	7,995 (70.1%)	16,724 (60.4%)
CAD	6,374 (15.3%)	2,104 (18.4%)	3,972 (14.3%)
Hyperlipidemia	15,161 (36.3%)	4,334 (38.0%)	9,568 (34.6%)
Diabetes	13,099 (31.3%)	4,172 (36.6%)	7,926 (28.6%)
CKD	9,271 (22.2%)	2,852 (25.0%)	5,333 (19.3%)
Heart failure	12,989 (31.1%)	3,605 (31.6%)	7,637 (27.6%)
EF, mean (s.d.), %	60 (13)	60 (12)	60 (13)
BSA, mean (s.d.), m^2^	1.86 (0.32)	1.85 (0.33)	1.85 (0.29)
TAPSE, mean (s.d.), mm	2.18 (1.19)	2.22 (1.41)	2.21 (1.24)
LVEDVI, mean (s.d.), ml m^−^^2^	53.82 (22.96)	53.81 (22.39)	53.42 (23.07)
LVESVI, mean (s.d.), ml m^−^^2^	23.03 (18.65)	22.52 (17.27)	22.63 (18.31)
LV mass, mean (s.d.), g	88.46 (30.55)	89.39 (31.49)	86.74 (30.53)
LVOT VTI, mean (s.d.), cm	0.22 (0.39)	0.23 (0.45)	0.23 (0.50)
RVOT VTI, mean (s.d.), cm	0.16 (0.36)	0.18 (0.61)	0.17 (0.51)
e′ lateral, mean (s.d.), cm s^−1^	0.10 (0.04)	0.10 (0.04)	0.10 (0.04)
e′ medial, mean (s.d.), cm s^−1^	0.08 (0.05)	0.08 (0.05)	0.08 (0.05)

CAD, coronary artery disease; CKD, chronic kidney disease; BSA, body surface area; TAPSE, tricuspid annular plane systolic excursion; LVEDVI, left ventricular end diastolic volume index; LVESVI, left ventricular end systolic volume index; LVOT VTI, left ventricular outflow tract velocity-time integral; RVOT VTI, right ventricular outflow tract velocity-time integral; e′ lateral, lateral mitral annular early diastolic velocity; e′ medial, septal mitral annular early diastolic velocity.

^a^Gender is calculated using the mode gender across all studies for a patient. Gender categories “other” and “unknown” were included in calculations but are not reported on this table.

**Fig. 3:**
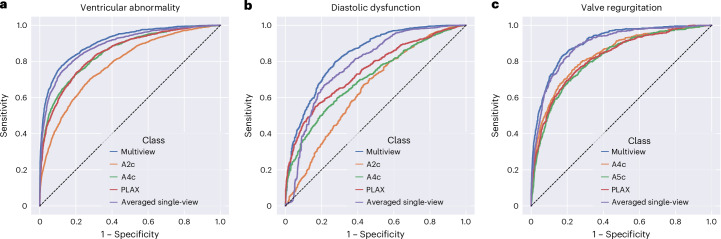
Performance of multiview versus single-view DNNs to predict three echo tasks in the UCSF test dataset. **a**–**c**, Receiver operating characteristic (ROC) curves showing overall performance of DNNs to predict LV/RV ventricular abnormality (**a**), diastolic dysfunction (**b**), and valve regurgitation (**c**) for both multiview DNNs (blue line) and single-view DNNs (orange, green, and red lines). Also shown is the ROC curve for the arithmetic average of the three single-view DNN outputs (purple line). The dotted line represents AUC of 0.5. Source data

In the held-out UCSF test dataset for LV/RV abnormality, the multiview DNN achieved an area under the receiver operating characteristic curve (AUC) of 0.907 (95% confidence interval (CI) 0.900–0.914) to detect LV/RV abnormality, and sensitivity and specificity were 0.810 and 0.840, respectively (Table 2). To provide comparison to single-view DNNs, we trained three separate X3D video-based single-view DNNs for each of the three echo views using the same dataset. The best single-view DNN for ventricular abnormality used the A4c view and achieved an AUC of 0.851 (95% CI 0.841–0.861), followed by PLAX with an AUC of 0.848 (95% CI 0.838–0.857) and then A2c with an AUC of 0.783 (95% CI 0.771–0.795) (Table 2 and Fig. 3). The multiview DNN for ventricular abnormality had a statistically significantly higher AUC than any single-view DNN and outperformed the best single-view A4c DNN AUC by 0.056 (Table 2). The ventricular abnormality multiview DNN F1 score was 0.695 (95% CI 0.679–0.710), which was higher than the F1 score for any single-view DNN (Table 2). As an additional comparator, we took the arithmetic average of the output scores from the three single-view DNNs and compared its discrimination performance to the multiview DNN. This is considered a “late fusion” approach and does not have the benefit of the DNN learning from multiple views simultaneously like our multiview DNN but is less computationally expensive and potentially easier to train compared to a multiview DNN. The average of three single-view DNNs had an AUC of 0.899 (95% CI 0.880–0.898), which was significantly higher than the AUC of any individual single-view DNN and statistically significantly lower than the AUC of the multiview DNN (Table 2 and Fig. 3; *P* < 0.001).

**Table 2:** Performance of multiview versus single-view DNNs for three separate echo tasks in the UCSF test dataset

DNN class	AUC	Sensitivity	Specificity	PPV	NPV	F1 score	*P* value
**LV/RV abnormality cohort, test dataset (*N*** = **6,279)**							
Multiview (A2c, A4c, PLAX)	0.907 (0.900–0.914)	0.810 (0.794–0.827)	0.840 (0.832–0.849)	0.609 (0.591–0.628)	0.935 (0.929–0.941)	0.695 (0.679–0.710)	ref.
Single-view A2c	0.783 (0.771–0.795)	0.642 (0.622–0.664)	0.772 (0.762–0.782)	0.464 (0.446–0.482)	0.875 (0.867–0.884)	0.539 (0.523–0.555)	<0.001
Single-view A4c	0.851 (0.841–0.861)	0.736 (0.716–0.753)	0.791 (0.781–0.800)	0.520 (0.501–0.537)	0.907 (0.899–0.914)	0.609 (0.593–0.624)	<0.001
Single-view PLAX	0.848 (0.838–0.857)	0.763 (0.744–0.78)	0.768 (0.758–0.778)	0.503 (0.486–0.521)	0.913 (0.905–0.920)	0.606 (0.591–0.621)	<0.001
Average of three single-view DNNs	0.889 (0.880–0.898)	0.780 (0.761–0.797)	0.840 (0.831–0.849)	0.599 (0.581–0.618)	0.925 (0.919–0.932)	0.671 (0.656–0.686)	<0.001
**Diastolic dysfunction cohort, test dataset (*N*** = **2,263)**							
Multiview (A2c, A4c, PLAX)	0.836 (0.821–0.851)	0.736 (0.718–0.755)	0.774 (0.747–0.801)	0.878 (0.864–0.893)	0.570 (0.544–0.596)	0.801 (0.787–0.815)	ref.
Single-view A2c	0.646 (0.625–0.667)	0.650 (0.631–0.670)	0.579 (0.549–0.612)	0.775 (0.756–0.793)	0.426 (0.400–0.452)	0.707 (0.691–0.722)	<0.001
Single-view A4c	0.708 (0.688–0.727)	0.712 (0.693–0.731)	0.596 (0.564–0.626)	0.797 (0.780–0.814)	0.482 (0.453–0.508)	0.752 (0.736–0.766)	<0.001
Single-view PLAX	0.749 (0.730–0.767)	0.777 (0.760–0.794)	0.596 (0.566–0.626)	0.811 (0.794–0.828)	0.546 (0.516–0.574)	0.794 (0.781–0.806)	<0.001
Average of three single-view DNNs	0.783 (0.771–0.794)	0.675 (0.665–0.686)	0.774 (0.759–0.791)	0.902 (0.894–0.910)	0.438 (0.424–0.452)	0.773 (0.765–0.780)	<0.001
**Valve regurgitation cohort, test dataset (*N*** = **4,148)**							
Multiview (A5c, A4c, PLAX)	0.904 (0.892–0.915)	0.826 (0.796–0.854)	0.829 (0.818–0.839)	0.395 (0.369–0.421)	0.972 (0.967–0.977)	0.534 (0.509–0.560)	ref.
Single-view A5c	0.816 (0.799–0.831)	0.763 (0.731–0.793)	0.716 (0.705–0.729)	0.267 (0.248–0.286)	0.957 (0.951–0.963)	0.395 (0.372–0.419)	<0.001
Single-view A4c	0.836 (0.821–0.852)	0.769 (0.738–0.798)	0.750 (0.739–0.762)	0.294 (0.273–0.315)	0.960 (0.954–0.966)	0.426 (0.400–0.448)	<0.001
Single-view PLAX	0.823 (0.806–0.839)	0.702 (0.667–0.737)	0.790 (0.779–0.801)	0.311 (0.287–0.335)	0.952 (0.945–0.958)	0.431 (0.405–0.457)	<0.001
Average of three single-view DNNs	0.891 (0.879–0.903)	0.791 (0.760–0.820)	0.829 (0.819–0.839)	0.385 (0.359–0.410)	0.967 (0.962–0.972)	0.518 (0.492–0.543)	0.018

All values are point estimates (95% CI, derived via bootstrap). We present *P* values comparing the AUC of multiview DNNs against each single-view DNN and the average of three single-view DNNs using the two-sided DeLong’s test with Bonferroni correction. *P* values lower than 1 × 10^−3^ are displayed as <0.001. ref., reference model for AUC comparisons.

NPV, negative predictive value.

Our second echo task was the novel composite task to identify the presence of diastolic dysfunction using non-doppler B-mode echo videos; these non-doppler videos are not used by cardiologists for interpretation of diastolic dysfunction making this a novel echo task. To develop the multiview DNN for diastolic dysfunction, we identified a cohort of 11,411 echo studies from 6,643 UCSF patients that had clinical interpretations of diastolic dysfunction in the echo report. The cohort had a mean age of 65 years (s.d. 16 years), and 50.3% were female (Table 1). The prevalence of any diastolic dysfunction in this cohort was 68.3%. In the held-out UCSF test dataset for diastolic dysfunction, the multiview DNN achieved an AUC of 0.836 (95% CI 0.821–0.851) to detect any diastolic dysfunction, and sensitivity and specificity were both ~0.76. (Table 2). As above, we also trained single-view X3D DNNs for diastolic dysfunction with each of the three views separately using the same dataset. The best single-view DNN performance to detect diastolic dysfunction used the PLAX view, with an AUC of 0.749 (95% CI 0.730–0.767) (Table 2), followed by A4c with an AUC of 0.708 (95% CI 0.688–0.727). The multiview DNN for diastolic dysfunction had a statistically significantly higher AUC than any single-view DNN and outperformed the best single-view PLAX DNN AUC by 0.087. The average of three single-view DNNs had an AUC of 0.783 (95% CI 0.771–0.794). This was significantly higher than the AUC for any individual single-view DNN and was statistically significantly lower than the AUC of the multiview DNN (Table 2 and Fig. 3; *P* < 0.001).

We then applied our multiview DNN architecture to detect the presence of any substantial valve regurgitation using color doppler echo videos. To develop a multiview DNN for valve regurgitation, we obtained 27,692 echo studies from 18,573 UCSF patients that also had clinical interpretations of valve regurgitation available in the clinical echo report. The cohort had a mean age of 61 years (s.d. 17 years), and 50% were female (Table 1). The prevalence of any valve abnormality in this cohort was 11.3%. In the held-out UCSF test dataset for valve regurgitation, the multiview DNN achieved an AUC of 0.904 (95% CI 0.892–0.915) to detect any substantial valve regurgitation, and sensitivity and specificity were both ~83% (Table 2). We also trained single-view X3D video-based DNNs for valve regurgitation with each of the three views separately using the same dataset. The best single-view DNN performance to detect valve regurgitation was obtained from the A4c view, with an AUC of 0.836 (95% CI 0.821–0.852; Table 2), followed by the PLAX view with an AUC of 0.823 (95% CI 0.806–0.839). The multiview DNN for valve regurgitation had a statistically significantly higher AUC than any single-view DNN and outperformed the best single-view A4c DNN AUC by 0.068 (Table 2). The average of three single-view DNNs had an AUC of 0.891 (95% CI 0.879–0.903), which was significantly higher than the AUC of any individual single-view DNN and was statistically significantly lower than the AUC of the multiview DNN (Table 2 and Fig. 3; *P* = 0.02).

### External validation of the multiview DNNs

To test the generalizability of our trained multiview DNN algorithms to external data from another institution, we measured the performance of our multiview DNNs on echos obtained from the Montreal Heart Institute (MHI) in Canada. This external validation dataset consisted of adult MHI echos acquired during 2022 (Extended Data Table 1). Labels were extracted from MHI clinical echo reports according to guideline criteria. Only linear measurements were available in MHI echos compared to volumetric criteria at UCSF for LV/RV abnormalities. In addition, the prevalence of cardiac abnormalities differed in the MHI test dataset versus UCSF: low LVEF (LV ejection fraction (EF)) was more common, and there was far less higher-grade diastolic dysfunction and abnormal RV function (Extended Data Table 1). We preprocessed MHI echo data and classified views using the same preprocessing algorithms. Upon reviewing the performance of the UCSF view classifier on 350 randomly selected MHI echo videos, the view classifier performed well across our target views with precision (positive predictive value (PPV)) of 100% for A4c, 83.3% for PLAX, 88.5% for A2c, and 81.8% for A5c, with a global accuracy of 79.14%.

On this MHI external validation dataset, the LV/RV abnormality multiview DNN achieved an AUC of 0.909 (95% CI 0.896–0.922; Table 3), and the valve regurgitation multiview DNN had an AUC of 0.924 (95% CI 0.890–0.954), both of which were comparable to DNN performance in the UCSF test dataset with overlapping 95% CIs. The diastolic dysfunction multiview DNN achieved an AUC of 0.791 (95% CI 0.765–0.817) in the MHI dataset, showing reasonable generalization with modest performance degradation compared to the UCSF test dataset (Table 3). In the MHI external dataset, multiview DNNs had higher AUCs than all single-view DNNs and the average of three single-view DNNs. However, for valve regurgitation, which was the smallest external validation cohort, the higher multiview DNN AUC was not statistically significantly higher than single-view DNNs (Table 3).

**Table 3:** External validation performance of multiview versus single-view DNNs on MHI data

DNN class	AUC	Sensitivity	Specificity	PPV	NPV	F1 score	*P* value
**LV/RV abnormality cohort, test dataset (*N*** = **1,650)**							
Multiview (A2c, A4c, PLAX)	0.909 (0.896–0.922)	0.837 (0.808–0.866)	0.799 (0.780–0.818)	0.614 (0.582–0.644)	0.928 (0.914–0.940)	0.708 (0.683–0.733)	ref.
Single-view A2c	0.787 (0.777–0.816)	0.604 (0.569–0.641)	0.812 (0.793–0.830)	0.550 (0.515–0.586)	0.843 (0.826–0.861)	0.576 (0.546–0.608)	<0.001
Single-view A4c	0.870 (0.854–0.885)	0.741 (0.705–0.775)	0.807 (0.788–0.826)	0.593 (0.561–0.627)	0.891 (0.874–0.906)	0.659 (0.632–0.686)	<0.001
Single-view PLAX	0.861 (0.843–0.876)	0.725 (0.689–0.761)	0.828 (0.809–0.845)	0.616 (0.583–0.650)	0.888 (0.871–0.903)	0.666 (0.638–0.694)	<0.001
Average of three single-view DNNs	0.892 (0.877–0.905)	0.802 (0.770–0.829)	0.800 (0.781–0.818)	0.604 (0.570–0.635)	0.914 (0.900–0.926)	0.689 (0.661–0.714)	0.031
**Diastolic dysfunction cohort, test dataset (*N*** = **766)**							
Multiview (A2c, A4c, PLAX)	0.791 (0.765–0.817)	0.502 (0.454–0.551)	0.852 (0.824–0.878)	0.694 (0.642–0.743)	0.719 (0.686–0.751)	0.582 (0.538–0.627)	ref.
Single-view A2c	0.647 (0.615–0.678)	0.603 (0.558–0.647)	0.599 (0.563–0.636)	0.501 (0.458–0.544)	0.693 (0.655–0.730)	0.547 (0.509–0.582)	<0.001
Single-view A4c	0.713 (0.683–0.743)	0.599 (0.551–0.646)	0.712 (0.679–0.747)	0.582 (0.536–0.629)	0.727 (0.692–0.763)	0.591 (0.553–0.630)	<0.001
Single-view PLAX	0.698 (0.666–0.728)	0.824 (0.789–0.860)	0.488 (0.450–0.523)	0.518 (0.481–0.555)	0.806 (0.764–0.846)	0.636 (0.602–0.668)	<0.001
Average of three single-view DNNs	0.743 (0.714–0.772)	0.404 (0.358–0.453)	0.852 (0.824–0.880)	0.646 (0.588–0.699)	0.681 (0.652–0.714)	0.497 (0.448–0.543)	0.024
**Valve regurgitation cohort, test dataset (*N*** = **303)**							
Multiview (A5c, A4c, PLAX)	0.924 (0.890–0.954)	0.750 (0.647–0.857)	0.886 (0.853–0.918)	0.500 (0.400–0.607)	0.959 (0.937–0.976)	0.600 (0.506–0.689)	ref.
Single-view A5c	0.878 (0.838–0.914)	0.725 (0.606–0.838)	0.840 (0.802–0.877)	0.408 (0.319–0.500)	0.953 (0.929–0.974)	0.523 (0.430–0.610)	0.658
Single-view A4c	0.881 (0.834–0.920)	0.725 (0.609–0.833)	0.817 (0.778–0.855)	0.377 (0.292–0.464)	0.951 (0.927–0.974)	0.496 (0.404–0.580)	0.100
Single-view PLAX	0.810 (0.746–0.870)	0.600 (0.486–0.725)	0.905 (0.875–0.935)	0.490 (0.375–0.612)	0.937 (0.913–0.960)	0.539 (0.432–0.641)	0.009
Average of three single-view DNNs	0.915 (0.875–0.948)	0.725 (0.615–0.833)	0.886 (0.856–0.919)	0.492 (0.390–0.606)	0.955 (0.932–0.975)	0.586 (0.488–0.678)	1.000

All values are point estimate (95% CI, derived via bootstrap). We present *P* values comparing the AUC of multiview DNNs against each single-view DNN and the average of three single-view DNNs using the two-sided DeLong’s test with Bonferroni correction. *P* values lower than 1 × 10^−3^ are displayed as <0.001.

### Multiview and single-view performance on individual components of composite endpoints

Composite endpoints, such as detection of any LV/RV abnormality, may inherently benefit from inclusion of multiple imaging views as each view contributes unique and complimentary information to the composite task. Indeed, we selected composite tasks because the multiview architecture may be best suited for such tasks. However, to investigate the benefit of our multiview DNN approach for more anatomically specific tasks, we trained single-view and multiview DNNs for individual components of our composite endpoints in the UCSF dataset: LV size, LVEF, RV function, RV size, mitral regurgitation, aortic regurgitation, and tricuspid regurgitation. As with previous experiments, single-view models were trained using videos from the same echo studies as multiview models but using only a single view (A4c, A2c, or PLAX for LV/RV abnormalities; A4c, A5c, or PLAX with color Doppler for valvular regurgitation). In the held-out UCSF test datasets for all seven tasks, the multiview DNNs consistently outperformed single-view DNNs, with the exception of tricuspid regurgitation (Table 4). The tricuspid regurgitation multiview DNN had a borderline non-significant difference with A4c DNN (Bonferroni-adjusted *P* = 0.057) and a non-significant difference with the PLAX DNN.

**Table 4:** Performance of multiview versus single-view DNNs for individual components of the composite labels

DNN class	AUC	Sensitivity	Specificity	PPV	NPV	F1 score
**LV size (*N*** = **6,256)**						
Multiview	0.902 (0.89 –0.914)	0.819 (0.789–0.848)	0.820 (0.812–0.829)	0.257 (0.238–0.277)	0.984 (0.98–0.986)	0.392 (0.368–0.415)
Single-view A2c ^(a)^	0.826 (0.809–0.843)	0.67 (0.63–0.706)	0.812 (0.803–0.82)	0.213 (0.194–0.231)	0.97 (0.965–0.974)	0.323 (0.298–0.346)
Single-view A4c ^(a,b)^	0.843 (0.827–0.858)	0.753 (0.72–0.787)	0.767 (0.758–0.776)	0.197 (0.182–0.213)	0.976 (0.972–0.98)	0.313 (0.291–0.335)
Single-view PLAX ^(b)^	0.859 (0.843–0.874)	0.733 (0.697–0.769)	0.811 (0.803–0.82)	0.228 (0.21–0.246)	0.976 (0.972–0.979)	0.348 (0.324–0.371)
**LVEF (*N*** = **6,256)**						
Multiview	0.942 (0.935–0.948)	0.823 (0.802–0.842)	0.906 (0.899–0.912)	0.616 (0.592–0.638)	0.966 (0.961–0.97)	0.704 (0.685–0.722)
Single-view A2c	0.879 (0.869–0.888)	0.807 (0.785–0.826)	0.783 (0.773–0.792)	0.405 (0.385–0.422)	0.957 (0.952–0.961)	0.539 (0.519–0.556)
Single-view A4c ^(c)^	0.900 (0.891–0.909)	0.84 (0.82–0.859)	0.791 (0.781–0.8)	0.423 (0.403–0.441)	0.964 (0.959–0.969)	0.563 (0.543–0.58)
Single-view PLAX ^(c)^	0.898 (0.888–0.907)	0.776 (0.755–0.797)	0.851 (0.843–0.859)	0.488 (0.467–0.508)	0.954 (0.949–0.959)	0.599 (0.58–0.618)
**RV function (*N*** = **6,256)**						
Multiview	0.931 (0.920–0.940)	0.906 (0.878–0.931)	0.821 (0.813–0.829)	0.219 (0.201–0.237)	0.994 (0.992–0.995)	0.353 (0.328–0.376)
Single-view A2c	0.814 (0.796–0.832)	0.778 (0.739–0.817)	0.703 (0.693–0.713)	0.127 (0.114–0.139)	0.983 (0.979–0.986)	0.218 (0.199–0.237)
Single-view A4c ^(d)^	0.896 (0.883–0.907)	0.872 (0.841–0.902)	0.733 (0.723–0.742)	0.154 (0.14–0.167)	0.99 (0.988–0.993)	0.261 (0.241–0.281)
Single-view PLAX ^(d)^	0.881 (0.865–0.894)	0.802 (0.765–0.837)	0.811 (0.803–0.819)	0.191 (0.173–0.208)	0.987 (0.984–0.989)	0.308 (0.283–0.332)
**RV size (*N*** = **6,256)**						
Multiview	0.897 (0.885–0.910)	0.835 (0.809–0.863)	0.805 (0.796–0.813)	0.259 (0.241–0.277)	0.984 (0.981–0.987)	0.395 (0.373–0.417)
Single-view A2c	0.705 (0.685–0.724)	0.665 (0.63–0.702)	0.618 (0.607–0.628)	0.124 (0.114–0.135)	0.958 (0.952–0.963)	0.209 (0.194–0.226)
Single-view A4c ^(e)^	0.861 (0.845–0.874)	0.79 (0.759–0.82)	0.764 (0.755–0.773)	0.215 (0.199–0.231)	0.978 (0.974–0.982)	0.338 (0.316–0.358)
Single-view PLAX ^(e)^	0.845 (0.827–0.861)	0.752 (0.718–0.785)	0.793 (0.784–0.801)	0.229 (0.212–0.247)	0.975 (0.971–0.979)	0.351 (0.328–0.373)
**Mitral regurgitation (*N*** = **4,148)**						
Multiview	0.930 (0.915–0.943)	0.842 (0.798–0.879)	0.866 (0.858–0.875)	0.268 (0.241–0.295)	0.990 (0.986–0.992)	0.407 (0.373–0.439)
Single-view A5c ^(f)^	0.868 (0.849–0.887)	0.794 (0.747–0.837)	0.767 (0.756–0.778)	0.165 (0.147–0.184)	0.985 (0.981–0.988)	0.274 (0.247–0.3)
Single-view A4c ^(f)^	0.858 (0.837–0.876)	0.768 (0.72–0.811)	0.771 (0.759–0.783)	0.163 (0.145–0.181)	0.983 (0.979–0.986)	0.269 (0.242–0.295)
Single-view PLAX	0.900 (0.884–0.915)	0.829 (0.788–0.868)	0.806 (0.796–0.816)	0.199 (0.177–0.22)	0.988 (0.985–0.991)	0.321 (0.29–0.349)
**Aortic regurgitation (*N*** = **4,148)**						
Multiview	0.929 (0.895–0.959)	0.862 (0.791–0.931)	0.884 (0.875–0.892)	0.105 (0.086–0.128)	0.998 (0.996–0.999)	0.188 (0.155–0.223)
Single-view A5c ^(g)^	0.873 (0.826–0.915)	0.661 (0.565–0.754)	0.913 (0.906–0.920)	0.108 (0.084–0.135)	0.994 (0.992–0.996)	0.186 (0.147–0.228)
Single-view A4c ^(h)^	0.786 (0.733–0.836)	0.692 (0.597–0.787)	0.742 (0.730–0.753)	0.041 (0.032–0.052)	0.993 (0.991–0.996)	0.077 (0.061–0.096)
Single-view PLAX ^(g,h)^	0.863 (0.821–0.902)	0.662 (0.561–0.758)	0.844 (0.834–0.853)	0.063 (0.048–0.079)	0.994 (0.991–0.996)	0.115 (0.088–0.142)
**Tricuspid regurgitation (*N*** = **4,148)**						
Multiview ^(i,j)^	0.864 (0.845–0.881)	0.782 (0.738–0.822)	0.814 (0.804–0.825)	0.221 (0.200–0.243)	0.982 (0.979–0.986)	0.345 (0.316–0.373)
Single-view A5c ^(k)^	0.813 (0.788–0.836)	0.733 (0.684–0.780)	0.766 (0.755–0.777)	0.175 (0.156–0.193)	0.977 (0.972–0.982)	0.282 (0.256–0.307)
Single-view A4c ^(i)^	0.884 (0.867–0.899)	0.863 (0.824–0.896)	0.743 (0.732–0.755)	0.184 (0.166–0.202)	0.988 (0.984–0.991)	0.304 (0.278–0.329)
Single-view PLAX ^(j,k)^	0.842 (0.819–0.864)	0.733 (0.688–0.775)	0.798 (0.788–0.809)	0.197 (0.176–0.217)	0.978 (0.974–0.982)	0.310 (0.283–0.337)

Multiview and single-view DNNs were trained for example individual components of the composite tasks in Table 2. Single-view models were trained using videos from the same studies as multiview models, but from a single view. All values are point estimate (95% CI, derived via bootstrap). Superscript letters indicate model pairs within each task with non-significant differences in AUC (*P* > 0.05), as determined using the two-sided DeLong’s test with Bonferroni correction for multiple comparisons. All model pairs within each task without superscripts had AUCs that were statistically significantly different from each other.

Furthermore, we examined how the composite task multiview DNNs performed within substrata of the UCSF test set for each component task. Overall for the ventricular abnormality, multiview DNN performance remained high (AUC > 0.90) for all abnormalities of LV/RV size or function (Extended Data Table 2). The diastolic dysfunction multiview DNN showed highest performance for grade 4 diastolic dysfunction, and the valve regurgitation multiview DNN showed highest performance for mitral regurgitation (Extended Data Table 2). Results in substrata of the MHI test dataset are shown in Extended Data Table 3.

### Additional examination of multiview DNN performance

Depending on the intended clinical application, the performance of a trained DNN can be modified to favor a higher sensitivity or specificity by selecting a different threshold. Extended Data Table 4 shows sensitivity-optimized and specificity-optimized multiview DNN performance in the UCSF test set achieved by fixing sensitivity or specificity at 0.80.

Multiview DNN performance was similar in strata of sex and age groups in UCSF (Extended Data Table 5). The performance of the multiview DNN for diastolic dysfunction also did not vary substantially when stratifying by LVEF (≥50% versus <50%) (Extended Data Table 6), suggesting that the DNN identified predictors of diastolic dysfunction independently of EF; this is important to confirm because of the association between reduced EF and diastolic dysfunction. Multiview DNN performance was similar when inference was run starting at a random frame of the video clip, rather than the first frame (Extended Data Table 7); although the valve model showed a slight shift in calibration with random frame data, overall discrimination did not change. Performance was also consistent across strata of echo machine manufacturer (Extended Data Table 8).

**Fig. 4:**
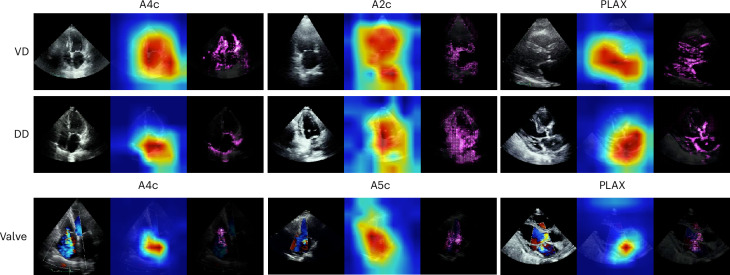
Grad-CAM and guided grad-CAM AI explainability applied to DNNs for three echo tasks. Grad-CAM and guided grad-CAM heat maps showing the class-weighted activations of the final convolutional layer in our single-view DNNs for (top to bottom) LV/RV abnormalities (VD), diastolic dysfunction (DD), and valvular regurgitation (Valve) using A2c, A4c, and PLAX views for VD and DD, and using A4c, A5c, and PLAX views for valve. For each panel, the left image is the original echo frame, the middle image is the grad-CAM, and the right image is the guided grad-CAM. Brighter red (grad-CAM) or pink (guided grad-CAM) areas indicate areas of greater importance for that DNN’s prediction from that frame.

**Extended Data Fig. 2:**
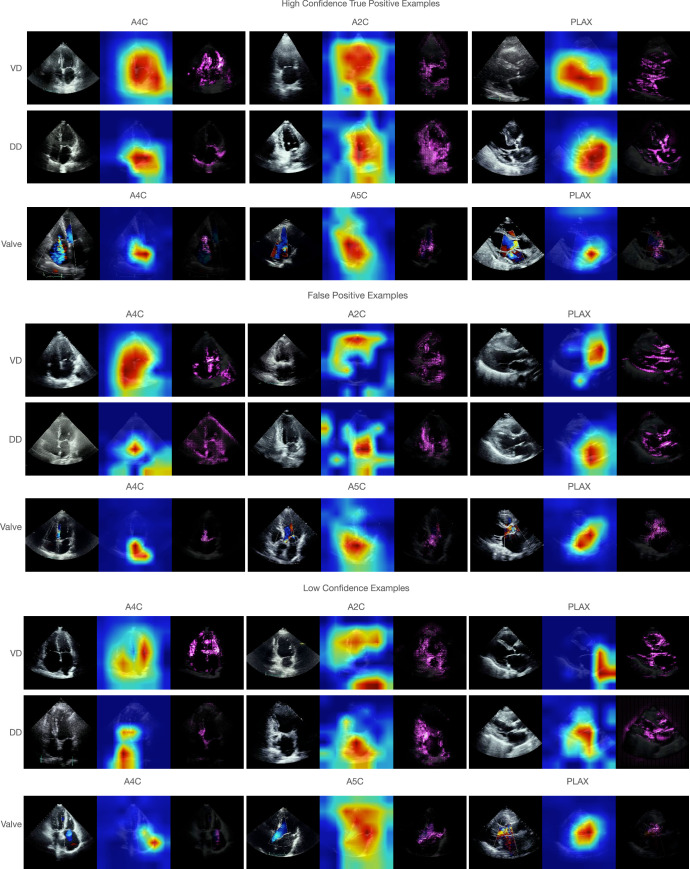
Grad-CAM and guided grad-CAM examples for false positive or low confidence DNN predictions. Grad-CAM and guided grad-CAM heatmaps showing the class-weighted activations of the final convolutional layer in our single-view DNNs for left/right ventricular abnormalities (VD) and diastolic dysfunction (DD) using apical two chamber (A2c), four chamber (A4c), and parasternal long axis (PLAX) views, and valvular regurgitation (valve) using A4c, apical 5 chamber (A5c) and PLAX views. These are shown for high confidence true positive examples, false positive examples and low confidence examples. For each panel, the left image is the original echo frame, the middle image is the Grad-CAM and the right image is the guided Grad-CAM. Brighter red (Grad-CAM) or pink (guided Grad-CAM) areas indicate areas of greater importance for that DNN’s prediction from that frame.

Explainable AI techniques allow for the identification of patterns within input echo videos that the DNN learned as being important to make its predictions, possibly highlighting physiologic associations with the target task. We used the guided grad-CAM technique (Gradient-weighted Class Activation Mapping)^23^ to visualize the pixels within images of a video that most strongly contribute to a prediction from a trained DNN (Fig. 4). We present both the grad-CAM and guided grad-CAM visualizations, as the former captures the general image regions influencing the prediction while the latter refines these maps to highlight pixel-level detail. Guided grad-CAM maps of the ventricular abnormality DNN tended to focus on both pixels of the right and left ventricular myocardium, with some preference for the left ventricle. Highlighted pixels for the diastolic dysfunction DNNs focused on the left atrium, but also of the LV myocardium and right atrium. Guided grad-CAM maps for the valvular regurgitation DNN highlighted valve tissue areas as well as the color doppler signal of valvular regurgitation, when present. It is important to appreciate, however, that current explainability techniques such as guided grad-CAM only provide a limited view into DNN function and thus should be considered accordingly. We also show example grad-CAM and guided grad-CAM images from false-positive and low-confidence predictions as comparators (Extended Data Fig. 2).

## Discussion

In this study, we developed and validated a multiview DNN architecture that integrates information from multiple input imaging views simultaneously, providing an opportunity to optimize AI algorithms for 3D medical imaging broadly. Across our primary demonstration tasks, our multiview DNN outperformed single-view DNNs improving overall discrimination by 0.06–0.09 AUC. In addition, we showed that by averaging outputs from three separately trained single-view DNNs, discrimination was significantly higher compared to any single-view DNN but also that the multiview DNN had statistically significantly better discrimination than the average of three single-view DNNs. Performance of the multiview DNNs remained robust across various substrata and generalized well to data from an external institution with modest performance degradation for diastolic dysfunction models; this may be partially explained by the difference in the prevalence of diastolic dysfunction at MHI. By integrating spatiotemporal information across multiple imaging video views, the multiview DNN can learn how the complementary information captured by each view relates to information in other views in a disease-specific manner, mirroring how physicians interpret complex medical imaging data. Considering multiple imaging views simultaneously, either through multiview DNN architectures or by averaging several single-view DNNs, provides improvements across various disease tasks, underscoring the value of a multiview paradigm when training AI models for medical imaging.

Medical imaging has always faced the challenge of capturing 2D tomographic slices of 3D anatomic structures. For imaging modalities such as echo, physicians are accustomed to reviewing and integrating findings from all available 2D views of a structure into a 3D (or higher dimensional) mental model before finalizing a final diagnostic impression. This challenge has prompted the development of technology such as 3D ultrasound^24^ which assists physician diagnosis by depicting 3D anatomic contours. However, 2D echo videos remain the primary diagnostic format, owing in part to limitations in 3D reconstruction, smoothing, artifacts, and spatial resolution^25^. Therefore, mental integration of information from multiple 2D imaging views remains the standard of care upon which most physician-reviewed echo diagnoses are made. To date, AI has primarily been used to analyze one 2D view at a time—from either images or videos—which limits an AI algorithm’s ability to learn disease-relevant information between views. Therefore, DNN architectures that can integrate information across multiple high-resolution views represent an important step toward maximizing AI performance in medical imaging.

In the case of echo, nearly every important diagnosis necessitates considering information from more than one view because the information from any single view tells only part of the story. For example, for the assessment of LV size or function, the standard A4c view captures the inferoseptal and anterolateral walls of the left ventricle, whereas the A2c view captures the anterior and inferior walls of the left ventricle (Fig. 1). It is not uncommon for the function of LV walls to appear completely normal in one view but for substantial dysfunction to be present in LV walls visible only in another view—this is called “regional myocardial wall motion abnormality” and is often caused by myocardial ischemia^26^. For composite echo tasks like LV/RV abnormality and diastolic dysfunction, our results suggest that the multiview DNN not only learns the best views to accomplish each subtask but also likely learns interrelated information between features from each view to achieve higher overall performance than any single-view DNN or the late-fusion average of three single-view DNNs. We would highlight, however, that the average of three single-view DNNs does provide a viable alternative to training a multiview DNN that improves performance beyond a single-view DNN and may be less computationally expensive.

As we observed, the performance gains provided by the multiview DNN architecture vary by task and would be expected to provide the greatest benefit for tasks that require simultaneous consideration of complementary inputs. It also may provide similar benefit for imaging modalities beyond echo. For example, we previously used the same DNN architecture to train multiview DNNs to estimate LV systolic function from multiple angiographic videos simultaneously^27^. We showed that for this “superhuman” task of estimating cardiac pumping function from left coronary artery angiogram videos, using the multiview DNN to consider multiple views simultaneously substantially outperformed DNNs that only considered one angiogram view. Corroborating our present results in echo videos, these results together suggest that for certain imaging tasks multiview DNNs trained with more than one input view can meaningfully improve performance over single-view DNNs alone. Future work should examine how multiview DNN architectures may assist other medical tasks or imaging modalities.

Prior efforts that used DNNs to analyze more than one echo view to accomplish a single task have most commonly done so by combining outputs from DNNs at a late stage. The simplest of these approaches takes the arithmetic average of the predictions from multiple separate single-view DNNs, similar to our reported average of three single-view DNNs. A slightly more complex approach combines representations derived from single-view DNNs just before a final network layer that outputs the final diagnosis^11,28^. These are both considered late fusion approaches as they fuse representations of separate single-view DNNs at a late stage. Late fusion approaches do not enable the DNNs to learn meaningful patterns or interactions between the views. Another approach that has been taken recently has been “view-agnostic” approaches that take embeddings from arbitrary numbers of views from an echo study to predict echo measurements or report characteristics^29,30^. In these approaches, all the available views in an echo study are considered together regardless of the target task. By contrast, our multiview DNN accepts the three input views most appropriate for the target task, and then performs multiple convolutions across the views with a mid-fusion approach. This enables the DNN to discover interrelated patterns between the raw videos from each view that most effectively accomplish the target task. Mid fusion approaches can theoretically increase predictive performance by integrating complementary and interrelated features from multiple input types more thoroughly^28,31^. Our results support this by showing that multiview DNNs outperform the late-fusion averaged single-view DNN performance for most tasks. However, the degree of performance boost provided by the multiview DNN will likely vary depending on the target task and the available training data. While our multiview DNN was significantly better for all of our composite tasks (Table 2), for the individual task of tricuspid regurgitation, the single-view DNNs for A4c and A5c performed similarly well, with A4c having a higher AUC (Table 4). It is also worth noting that the PLAX view does not directly visualize tricuspid regurgitation; therefore, the PLAX DNN likely discriminates tricuspid regurgitation based on correlative echo features. Interestingly, in our external validation MHI dataset, the valve regurgitation single-view and multiview DNN AUCs were all higher than observed in our internal UCSF test dataset; in this context, the higher multiview DNN AUC was not statistically significantly higher than the single-view DNN’s AUCs (Table 4). Overall, our multiview DNN approach and results underscore the concept that for certain medical imaging tasks, optimal AI model training involves considering multiple views simultaneously.

Previously published DNNs for similar echo tasks have typically used single-view models and have not attempted composite echo tasks. Multiview DNNs offer the possibility to accomplish these higher-level composite tasks that may be less ideal to perform using a single view because the necessary information is not contained by any single view. Our work examines DNN performance for composite echo tasks—such as any LV/RV dysfunction or any valve regurgitation—using a single end-to-end multiview DNN. To classify LV dysfunction, ref. ^7^ trained a video-based model for the single A4c view and reported an AUC of 0.97 to detect reduced LV function, and ref. ^16^ reported similar results with an earlier image-based DNN classifier. Our LV/RV abnormality multiview DNN identifies abnormal LV function as one component of its composite task but simultaneously also interrogates the three other abnormalities of highest clinical relevance for the left ventricle and right ventricle across three echo views. Together, the multiview DNNs for LV/RV function and valve regurgitation could be clinically used to triage echos as being broadly normal or abnormal, with abnormal-predicted echos receiving more urgent physician review. Single-view DNNs have previously been used for many prior disease identification tasks like amyloid and hypertrophic cardiomyopathy^12,16^, wall motion abnormalities^13^, LV hypertrophy^12^, cardiac constriction and restriction^14^, myocardial strain^16,32^, RV function^33^, and atrial septal defect detection^34^. Our results suggest that some of these tasks may benefit from a multiview DNN approach, formulated either as individual or composite tasks.

Our study is best interpreted in the context of its limitations. The primary technical limitation of training multiview DNNs is the higher input dimensionality compared to single-view DNNs, which has several implications. First, substantially greater amount of data are often required to adequately train a multiview DNN compared to single-view DNNs for a similar task. In addition, only studies containing all three views needed by the multiview DNN can be used in either training or inference. In our experiments, the proportion of studies excluded due to missing views was 10.9% for LV/RV abnormality, 6.1% for diastolic dysfunction, and 56.1% for valve regurgitation (mitral valve, aortic valve, and tricuspid valve). This limitation may make it harder to train multiview DNNs for rare diseases. Second, given their high input dimensionality, multiview DNNs require greater computational capacity and graphical processing unit memory to train. In this context, the average of the three single-view DNN outputs may provide an attractive alternative requiring lower data and computational capacity despite the lower overall performance compared to a multiview DNN. Multiview DNNs are best suited for specific tasks or clinical settings where it is imperative to consider the information from multiple input views simultaneously. Another limitation is that our definition of “substantial valvular regurgitation” does not include the pulmonic valve; we chose not to include this because it requires a dedicated echo view that is often not clearly visualized in echo and because pulmonary regurgitation has lower clinical consequence compared to other valvular regurgitation. Discussion points are continued in the Supplementary Information.

In conclusion, we describe a general-purpose multiview DNN architecture and demonstrate that it achieves substantial performance improvements compared to single-view DNNs across a range of cardiac echo tasks. If confirmed by future work applying multiview DNNs to other imaging modalities and diseases and in multi-institutional settings, the multiview approach provides a powerful paradigm to train multiview optimized AI models for medical imaging.

## Methods

### Cohort selection and data sources

Our UCSF echocardiogram dataset comprised studies acquired on adult patients at UCSF between 2012 and 2020. These raw imaging data are linked with structured diagnoses and measurement data, including quantitative and qualitative measures adjudicated by level 3 echocardiographers at the UCSF echo lab. Measurement of RV parameters was standard practice in the UCSF echo lab during the study period; RV size and function labels were present for the majority of studies. Pixel data were extracted from the Digital Imaging and Communications in Medicine format, and the echo imaging region (cone) was identified by generating a mask of pixels with intensity changes over time. We then applied erosion and dilation operations to remove smaller moving elements such as electrocardiogram waveforms. Videos were then cropped to the smallest square that contained the entire echo imaging cone and resized to 224 × 224 pixels. All analyses excluded the following study types: transesophageal, intracardiac, and stress tests of any kind. This study was reviewed and received approval from the Institutional Review Boards of UCSF and the University of Montreal which waived the need to obtain informed consent in the setting of this minimal-risk retrospective record research.

To train the video-based view classifier, 6,549 echocardiograms from 1,437 patients were manually labeled. The 20 most common views were labeled, with the remainder classified as “other.” To simulate real-world data flow where all clinically obtained videos would undergo view classification, “other” was included as a trained class for the DNN. Views in the “other” class included view from transesophageal echo, color compare, split screen, among others. By training the DNN view classifier to classify 21 distinct echo views, more views than previously published image-based view classifiers, this served to reduce input variance into the DNN; this allows for discrimination, for example, between standard depth PLAX views, PLAX zoomed on the left atrium, or PLAX zoomed on the aortic valve^16^. This view classifier DNN achieved a mean AUC of 0.972 across the 21 classes (Extended Data Fig. 1). We trained a similar video-based DNN to classify the presence or absence of color doppler within the echo video which achieved an AUC of 0.991 (Extended Data Fig. 1).

These view/doppler-classifier DNNs automatically identified input echo videos comprising the predefined view and doppler combination for each task. We then applied these view/doppler DNN classifiers to all UCSF echos to identify the specific echo videos needed for training and validation for each of the three demonstration tasks. For the ventricular abnormality and diastolic dysfunction multiview DNNs, the three input echo views used were non-doppler A4c, A2c, and PLAX views; and for valve regurgitation, the input echo views were color-doppler A4c, A5c, and PLAX views. To ensure a fair comparison between single-view and multiview models, we first excluded all studies that were missing any required views. Both model types were trained and evaluated on videos from the same studies, the only difference being whether a model received a single view or three views as input.

For the ventricular abnormality dataset, we included all patients in our dataset with measures of ventricular function, comprising 36,023 echo studies from 11,334 patients. Of these, 2,907 patients were identified as having a ventricular abnormality, defined as having any abnormal measure of EF, LV size, RV size, or RV function. Ventricular abnormality was defined as positive if there was any abnormality in LV/RV size or function. LV size abnormality was defined as greater than mild dilation, which was LV end diastolic volume index of >86 ml m^−2^ for men or >70 ml m^−2^ for women. LV functional abnormality was defined as LVEF < 50%, measured by Simpson’s biplane approach^19^. RV size abnormality was defined as moderately increased or greater, and abnormal RV function was defined as moderately decreased or greater^19^.

For diastolic dysfunction, we included all patients with measures of diastolic dysfunction, comprising 11,341 echo studies from 6,649 patients. Diastolic dysfunction was defined as any diastolic dysfunction (grades 1–4) as determined by ASE guidelines^20,21^. Of these, we identified 4,774 patients showing any diastolic dysfunction measure above grade 0. Grades 1–2 included any annotation of abnormal or impaired relaxation, or increased filling pressure. Grade 3 included all pseudonormal diastolic function, and grade 4 included restrictive diastolic function.

For valve regurgitation, we included all patients with measures of valve regurgitation, comprising 27,652 echo studies from 18,533 patients. Any substantial valve regurgitation was defined as moderate or greater regurgitation in any of the mitral, tricuspid, or aortic valves according to ASE guidelines^22^. Of these, we identified 969 patients with mitral valve regurgitation, 329 patients with aortic valve regurgitation, and 1,299 patients with tricuspid valve regurgitation.

Our external validation dataset consisted of all echos acquired at the MHI in 2022 on patients over the age of 18 years. These studies were similarly linked to the clinical echo reports from which we obtained qualitative and quantitative reference labels. The MHI echo lab uses linear measurements (compared with volumetric measurements at UCSF), which resulted in our needing to use linear measurements to define certain “abnormal” findings at MHI, as described below. For LV function, we labelled studies with an EF < 50% to be abnormal. Studies with a basal RV size measurement >4.4 cm were labelled abnormal. Studies with a basal LV size measurement of 6.3 cm for men or 5.6 cm for women were defined to be abnormal. For RV function, abnormality was defined as more than moderately decreased function (qualitative). Diastolic function grade labels were available in MHI and followed ASE guidelines^20,21^. Measurement of RV parameters was standard practice in the MHI echo lab during the study period. MHI echos were processed using identical preprocessing as described above. Preprocessing and view classification performance in MHI were assessed by randomly reviewing 350 MHI clips labelled by the UCSF-trained view classifier. The view classifier performed well across our target views with precision (PPV) of 100% for A4c, 83.3% for PLAX, 88.5% for A2c, and 81.8% for A5c, with a global accuracy of 79.14%. We deployed our trained multiview DNNs on all MHI echos that had pertinent diagnostic labels and the three predefined views for each task. We then ran inference using the three multiview DNNs on all MHI studies containing the appropriate views.

### DNN architectures

For view classification, doppler detection, and all single-view models, we chose as our video-based DNN backbone the 3D convolutional neural network X3D-Medium (X3D-M), from the family of X3D architectures^15^. We tested other video-based DNN backbones, including R2-1D and the transformers ViViT, MoviNet, and STAM, and found X3D the most performant backbone overall. X3D-M had the additional benefit of being relatively lightweight compared to these other models, with 3.8 million parameters compared to R2-1D’s 33.3 million. This computational efficiency enabled faster training, larger batch sizes, and, eventually, expansion of the architecture to incorporate multiview video input.

For our multiview analysis, we developed a bespoke architecture to integrate multiple views with an enhanced mid-fusion strategy. First, the DNN passes each view, consisting of a 64 × 224 × 224 × 3 video, through five convolutional blocks consisting of 3D convolutions, batch normalization, and a rectified linear unit non-linearity, producing temporally and spatially reduced embeddings of shape (B, C, T, H, W) representing batch, channel, time, height, and width. These first five blocks are unchanged from the original X3D-M architecture^15^. The resulting embeddings are stacked along a new view dimension, V, to produce a tensor of shape (B, C, V, T, H, W). This tensor is then flattened across the time, height, and width dimensions, resulting in a tensor of shape (B, C, V, THW). A sixth convolutional block (following the same convolution, batch normalization, rectified linear unit format) performs a 2D convolution across the view and combined spatiotemporal dimensions to fuse information across the views. The tensor is then reshaped to (B, CT, V, H, W) and passed to the final convolutional block. This final block expands the number of channels by a factor of 128 as it performs a 3D convolution across view, height, and width. This step is crucial for deep integration of spatiotemporal information across views. The resulting tensor is then reshaped to (B, CV, T, H, W), and we perform average pooling on time, height and width dimensions before passing the final tensor through a fully connected layer and a decision head. The final multiview DNN has 230 million parameters.

### DNN training

All DNNs were developed and trained in Python (version 3.8.8) using the Pytorch library^35^ (version 1.8.8). Training of single-view DNNs took approximately 3 h; training of multiview DNNs took approximately 30–50 h on dual NVIDIA Quadro RTX 8000. For binary classification, we used a sigmoid decision head and the binary cross entropy loss, and for multiclass classification, we used a softmax decision head and the cross entropy loss function. For each of the three demonstration echo tasks separately, the data were divided into training/development/test datasets specific to that task in a 70/15/15 ratio, split by patient. The development dataset was used during training for learning rate decay scheduling and selection of the final models. The test dataset was held out from any model training or development and used to calculate evaluation metrics once the final DNNs for each task were trained.

Input videos to the DNN consisted of the first 64 frames of the video. Videos shorter than 64 frames were padded with empty frames. Echo video frame rates were 33 ± 17 frames per second. We did not normalize frame rates in the final training process, as this was tried and did not improve performance. The view classifier DNN was trained for 1,000 epochs starting with a learning rate of 0.01 and reducing learning rate using a factor of 0.5 with a patience of 50 and a loss threshold of 0.01. A separate doppler detection algorithm was trained on the same data with the same parameters. After training, the checkpoint achieving the lowest loss on the validation set was selected as the final DNN.

To train the single- and multiview DNNs, we used a standard hyperparameter sweep paradigm to allow all models to achieve their optimal performance and enable comparison. We performed separate hyperparameter sweeps over identical ranges of learning rate, threshold, and patience for learning rate decay for each task and view combination. For each sweep, we sampled learning rate from a log-uniform distribution between 1 × 10^−6^ and 5 × 10^−2^. For learning rate decay, we used the ReduceLRonPlateau scheduler monitoring validation loss with a 5% threshold. The scheduler patience was randomly sampled from (3, 5, 7, 10) and factor from (0.3, 0.5, 0.7). All models were trained for a total of 50 epochs without early stopping over 40 sweep trials with fixed random seeds for reproducibility. All models were trained on a single fixed data split for each task dataset. Both the input data size and model parameter sizes are substantially larger for the multiview DNNs resulting in increased training time for multiview models. We used the stochastic gradient descent optimizer (momentum = 0.9; weight decay = 0.0001) for all training runs. Training data were augmented gently using random resized crop between 0.95 and 1.0, color jitter between 0.8 and 1.2, and random rotation between −5 and 5 degrees. After training, the checkpoint achieving the highest AUC on the development set was selected as the final DNN.

All DNNs were evaluated using a combination of AUC and sensitivity/specificity at an optimal threshold defined as the threshold at which the geometric mean of sensitivity and specificity was maximal. Multiclass DNNs were evaluated using the mean AUC per class.

### Explainability analysis

To examine the features from the input video that contributed to the DNN predictions, we used a custom adaptation of the guided class-discriminative gradient class activation mapping algorithm (guided grad-CAM) to examine single-view model performance^23^. This adapted the 2D implementation to expand the dimensionality to accommodate 3D video data. This provides an approximation of what echo video pixels the multiview DNN model may be focusing on, with the caveat that these are single-view approximations. Representative videos were chosen for high diagnostic quality and confident disease-positive predictions (>0.95), and the adapted guided grad-CAM approach was used to generate heat maps corresponding to the pixels that most strongly contributed to that DNN’s prediction. In addition to the guided grad-CAM, we also generated standard grad-CAM maps to provide a course, class-discriminative localization of relevant regions, while guided grad-CAM highlights fine-grained pixel-level features.

### Statistical analysis

All continuous values are presented as mean ± 95% CI. For binary classification DNNs, the output of the final sigmoid function was a score ranging [0–1]. We report performance metrics using a default threshold for each DNN that was selected to maximize the F1 score on the development dataset for each task^36^. For the sensitivity/specificity-optimized sensitivity analysis, DNN performance metrics are reported at thresholds in the test dataset that fix sensitivity or specificity at 0.800. Statistical analyses were conducted in Python using pandas 2.3.0, numpy 1.26.4, scikit-learn 1.6.1, statsmodels 0.14.5, and MLstatkit 0.1.7.

The multiclass view classification DNN output consists of 21 continuous values ranging [0–1] with the predicted view corresponding to the maximum of the 21 values. For all test datasets, we present the AUC, sensitivity, specificity, and F1 score. CIs were derived by sampling the test set with replacement for 1,000 iterations to obtain 5th and 95th percentile values.

Differences in AUCs were tested using DeLong’s test^37^; in settings of multiple comparisons, Bonferroni correction was performed by adjusting the *P* values while retaining the threshold for significance at <0.05 (ref. ^38^). DeLong’s test was implemented using the MLstatkit package (version 0.1.7) in Python, and Bonferroni correction was implemented using the statsmodels package (version 0.14.5) in Python. Statistical significance was defined as *P* < 0.05.

For stratified analyses, we computed performance metrics for each DNN separately on strata of the test sets regarding age, gender, and disease subtypes. We defined disease substrata as those studies meeting previously described criteria for each abnormality compared to studies without criteria for abnormalities within each of the three echo tasks separately.

### Reporting summary

Further information on research design is available in the Nature Portfolio Reporting Summary linked to this article.

## Supplementary information

Supplementary Information Discussion and references.

Reporting Summary

## Source data

Source Data Fig. 3 Source data for ROC plots.

## Data Availability

The complete development dataset used in this study is derived from patient care and thus is not made publicly available due to data privacy concerns. A limited deidentified dataset to demonstrate algorithm functionality is available at https://www.openicpsr.org/openicpsr/project/241296. Reasonable requests for collaboration using the data can be made from the authors, as feasible and permitted by the Regents of the University of California.

## Extended data

**Extended Data Table 1:** Montreal Heart Institute cohort characteristics

Number (%)			
Characteristic	Ventricular Abnormality	Diastolic Dysfunction	Valve Regurgitation
Patients	1618	762	303
Age, mean (SD)	65(16)	61(16)	65 (16)
Female (%)	604 (%)	285 (%)	96 (31.7%)
Male (%)	1014 (%)	477 (%)	207 (68.3%)
Echo studies	1650	766	303

Characteristic	UCSF	MHI
**LV/RV Abnormality (Composite)**	23.5%	30.1%
Abnormal LV size	7.1%	5.8%
Abnormal LV ejection fraction	15.5%	23.6%
Abnormal RV size	7.5%	9.1%
Abnormal RV function	5.3%	1.3%
**Diastolic Dysfunction (Composite)**	69.0%	40.1%
DD Grade 1-2	50.0%	37.1%
DD Grade 3	15.8%	3.0%
DD Grade 4	3.2%	0.0%
**Valve Regurgitation (Composite)**	11.9%	13.2%
Mitral Regurgitation	5.5%	8.9%
Aortic Regurgitation	1.6%	1.3%
Tricuspid Regurgitation	6.3%	5.3%

Legend: (SD) Standard deviation. Abnormalities are assessed at the level of each individual echocardiogram study. Legend: (LV) Left ventricle, (RV) Right ventricle, (DD) Diastolic dysfunction, (MR) Mitral regurgitation, (AR) Aortic regurgitation, (TR) Tricuspid regurgitation; *moderate in severity or greater, per American Society of Echocardiography guidelines.

**Extended Data Table 2:** Performance of multi-view DNNs in disease strata in the UCSF test dataset

Ventricular Abnormality Type (value (95%CI))	AUC	Sensitivity	Specificity	F1
**Abnormal LV Size** (>86 ml/m2 for males, >70 ml/m2 for females) (N=4888)	0.965 (0.946–0.983)	0.929 (0.880–0.975)	0.840 (0.831–0.848)	0.170 (0.143–0.198)
**Abnormal LV Function** (LVEF <50%) (N=5692)	0.949 (0.943–0.955)	0.901 (0.885–0.918)	0.840 (0.831–0.848)	0.652 (0.633–0.67)
**Abnormal RV Size** (≥moderately enlarged) (N=5262)	0.903 (0.890–0.915)	0.806 (0.774–0.837)	0.840 (0.831–0.849)	0.463 (0.438–0.489)
**Abnormal RV Function** (≥moderately decreased) (N=5125)	0.950 (0.939–0.959)	0.891 (0.861–0.92)	0.840 (0.831–0.849)	0.417 (0.389–0.441)
**Diastolic Dysfunction by grade** (value (95%CI))	**AUC**	**Sensitivity**	**Specificity**	**F1**
Grade 1–2 (N=1765)	0.836 (0.821–0.853)	0.734 (0.71–0.756)	0.777 (0.751–0.805)	0.784 (0.767–0.8)
Grade 3 (N=1022)	0.836 (0.817–0.856)	0.736 (0.696–0.776)	0.777 (0.753–0.804)	0.677 (0.644–0.708)
Grade 4 (N=1088)	0.843 (0.824–0.862)	0.742 (0.707–0.778)	0.777 (0.751–0.803)	0.704 (0.674–0.732)
**Regurgitation by valve** (value (95%CI))	**AUC**	**Sensitivity**	**Specificity**	**F1**
Aortic Valve (N=3719)	0.891 (0.863–0.918)	0.769 (0.677–0.855)	0.829 (0.818–0.839)	0.135 (0.106–0.162)
Mitral Valve (N=3882)	0.943 (0.932–0.953)	0.904 (0.868–0.933)	0.829 (0.819–0.839)	0.389 (0.361–0.423)
Tricuspid Valve (N=3916)	0.885 (0.868–0.902)	0.790 (0.747–0.831)	0.829 (0.819–0.839)	0.378 (0.346–0.408)

(DNN) Deep Neural Network, (LV) Left ventricle, (RV) Right ventricle, (AUC) Area under the receiver operating characteristic curve, (CI) Confidence Interval. All values are point estimate (95% confidence interval, derived via bootstrap).

**Extended Data Table 3:** Performance of multi-view DNNs in disease strata in the Montreal Heart Institute (MHI) dataset

Ventricular Abnormality Type (value (95%CI))	AUC	Sensitivity	Specificity	F1
Abnormal LV Size (N=95+, 1154-)	0.973 (0.961–0.985)	0.937 (0.896–0.975)	0.807 (0.789–0.825)	0.437 (0.387–0.483)
Abnormal LV Function (N=390+, 1154-)	0.930 (0.917–0.942)	0.869 (0.840–0.896)	0.807 (0.788–0.826)	0.712 (0.684–0.739)
Abnormal RV Size (N=150+, 1154-)	0.830 (0.798–0.859)	0.693 (0.626–0.752)	0.807 (0.787–0.827)	0.436 (0.388–0.483)
Abnormal RV Function (N=21+, 1154-)	0.960 (0.906–0.996)	0.952 (0.857–1.000)	0.807 (0.788–0.826)	0.152 (0.101–0.201)
**Diastolic Dysfunction by grade** (value (95%CI))	**AUC**	**Sensitivity**	**Specificity**	**F1**
Grade 1-2 (N=284+, 459-)	0.783 (0.753–0.809)	0.479 (0.430–0.528)	0.852 (0.824–0.879)	0.557 (0.510–0.601)
Grade 3+ (N=23+, 459-)	0.893 (0.832–0.941)	0.783 (0.625–0.926)	0.852 (0.824–0.877)	0.330 (0.229–0.426)
**Regurgitation by Valve*** (value (95%CI))	**AUC**	**Sensitivity**	**Specificity**	**F1**
Aortic Valve (N=4+, 263-)	0.899 (0.800–0.992)	0.500 (0.000–1.000)	0.886 (0.852–0.916)	0.111 (0.000–0.231)
Mitral Valve (N=27+, 263-)	0.941 (0.902–0.973)	0.852 (0.719–0.960)	0.886 (0.856–0.916)	0.575 (0.456–0.674)
Tricuspid Valve (N=16+, 263-)	0.930 (0.874–0.975)	0.750 (0.563–0.929)	0.886 (0.852–0.917)	0.414 (0.280–0.538)

(DNN) Deep Neural Network, (LV) Left ventricle, (RV) Right ventricle, (AUC) Area under the receiver operating characteristic curve, (CI) Confidence Interval. *For the valvular regurgitation MHI cohort, we additionally report positive (+) and negative (-) studies in each substratum due to low total counts for valvular regurgitation in this external validation dataset. All values are point estimate (95% confidence interval, derived via bootstrap).

**Extended Data Table 4:** Performance of multi-view DNNs at sensitivity- and specificity-optimized thresholds in the UCSF test dataset

DNN class	Sensitivity-Optimized		Specificity-Optimized	
	Fixed Sensitivity	Specificity	Sensitivity	Fixed Specificity
**Left/Right Ventricular Abnormality cohort, test set (N=6279)**				
LVRV Multi-view DNN (value (95%CI))	0.800 (0.783–0.817)	0.851 (0.843–0.859)	0.837 (0.821–0.853)	0.800 (0.790–0.809)
**Diastolic dysfunction cohort, test set (N=2214)**				
Diastolic Dysfunction Multi-view DNN (value (95%CI))	0.800 (0.782–0.817)	0.721 (0.692–0.749)	0.691 (0.671–0.710)	0.800 (0.773–0.825)
**Valve regurgitation cohort, test set (N=4148)**				
Valve Regurgitation Multi-view DNN (value (95%CI))	0.800 (0.770–0.832)	0.845 (0.836–0.855)	0.850 (0.822–0.877)	0.800 (0.789–0.810)

The threshold of each trained Multi-view DNN was selected to fix sensitivity or specificity to 0.800 in the test dataset as indicated. (DNN) Deep Neural Network, (CI) Confidence Interval. All values are point estimate (95% confidence interval, derived via bootstrap).

**Extended Data Table 5:** DNN performance by demographic strata in the UCSF test dataset

Left/Right Ventricular Abnormality (value (95%CI))	AUC	Sensitivity	Specificity	F1
Males (N=3002)	0.904 (0.894–0.914)	0.826 (0.805–0.847)	0.81 (0.796–0.824)	0.725 (0.707–0.744)
Females (N=3241)	0.907 (0.894–0.918)	0.79 (0.761–0.817)	0.864 (0.853–0.875)	0.655 (0.632–0.678)
Age 18–60 (N=2550)	0.912 (0.900–0.922)	0.834 (0.811–0.861)	0.84 (0.826–0.854)	0.723 (0.701–0.745)
Age 61- (N=3695)	0.905 (0.895–0.915)	0.795 (0.773–0.818)	0.84 (0.828–0.851)	0.676 (0.655–0.695)
**Diastolic Dysfunction** (value (95%CI))	**AUC**	**Sensitivity**	**Specificity**	**F1**
Males (N=1069)	0.819 (0.795–0.843)	0.718 (0.692–0.745)	0.758 (0.719–0.799)	0.790 (0.769–0.809)
Females (N=1145)	0.851 (0.832–0.872)	0.755 (0.729–0.780)	0.787 (0.750–0.820)	0.812 (0.793–0.830)
Age 18–60 (N=721)	0.830 (0.805–0.854)	0.543 (0.490–0.592)	0.876 (0.851–0.901)	0.624 (0.579–0.665)
Age 61- (N=1493)	0.752 (0.726–0.780)	0.779 (0.760–0.797)	0.589 (0.540–0.644)	0.838 (0.824–0.851)
**Valve Regurgitation** (value (95%CI))	**AUC**	**Sensitivity**	**Specificity**	**F1**
Males (N=2088)	0.903 (0.887–0.919)	0.849 (0.815–0.885)	0.821 (0.806–0.835)	0.564 (0.532–0.598)
Females (N=2060)	0.903 (0.886–0.918)	0.795 (0.748–0.839)	0.837 (0.822–0.850)	0.498 (0.454–0.535)
Age 18–60 (N=1715)	0.926 (0.911–0.941)	0.808 (0.756–0.857)	0.879 (0.865–0.893)	0.552 (0.506–0.598)
Age 61- (N=2433)	0.888 (0.872–0.903)	0.835 (0.799–0.869)	0.792 (0.777–0.807)	0.526 (0.493–0.557)

(DNN) Deep Neural Network, (LV) Left ventricle, (RV) Right ventricle, (AUC) Area under the receiver operating characteristic curve, (CI) Confidence Interval. All values are point estimate (95% confidence interval, derived via bootstrap).

**Extended Data Table 6:** Multi-view DNN performance for diastolic dysfunction detection in patients stratified by ejection fraction in the UCSF test dataset

Diastolic Dysfunction (value (95%CI))	AUC	Sensitivity	Specificity	F1
Multi-view DNN, EF ≥ 50% (N=1973)	0.834 (0.817–0.849)	0.722 (0.702–0.743)	0.78 (0.754–0.805)	0.789 (0.773–0.804)
Multi-view DNN, EF < 50% (N=241)	0.812 (0.734–0.883)	0.826 (0.78–0.87)	0.647 (0.511–0.786)	0.877 (0.845–0.905)

(DNN) Deep Neural Network, (EF) Ejection Fraction, (AUC) Area under the receiver operating characteristic curve, (CI) Confidence Interval. All values are point estimate (95% confidence interval, derived via bootstrap).

**Extended Data Table 7:** Multi-view DNN performance on random start frames of echo video clips

DNN class	AUC	Sensitivity	Specificity	PPV	NPV	F1
**Left/Right Ventricular Abnormality cohort, test dataset (N=6279)**						
LV-RV Multi-view DNN (value (95% CI))	0.906 (0.899–0.914)	0.793 (0.778–0.810)	0.858 (0.850–0.867)	0.632 (0.614–0.651)	0.931 (0.925–0.937)	0.703 (0.688–0.719)
**Diastolic Dysfunction cohort, test dataset (N=2263)**						
Diastolic Dysfunction Multi-view DNN (value (95% CI))	0.839 (0.823–0.853)	0.734 (0.716–0.752)	0.772 (0.746–0.798)	0.877 (0.862–0.892)	0.568 (0.541–0.595)	0.799 (0.785–0.812)
**Valve Regurgitation cohort, test dataset (N=4148)**						
Valve Regurgitation Multi-view DNN (value (95% CI))	0.907 (0.895–0.917)	0.939 (0.920–0.956)	0.679 (0.667–0.692)	0.284 (0.266–0.303)	0.988 (0.984–0.991)	0.437 (0.414–0.459)

(DNN) Deep Neural Network, (AUC) Area under the receiver operating characteristic curve, (PPV) Positive Predictive Value, (NPV) Negative Predictive Value, (LV) Left Ventricle, (RV) Right Ventricle, (CI) Confidence Interval. All values are point estimate (95% confidence interval, derived via bootstrap).

**Extended Data Table 8:** Multi-view DNN performance stratified by echo machine manufacturer

Left/Right Ventricular Abnormality (value (95%CI))	AUC	Sensitivity	Specificity	PPV	NPV	F1
All data (N=6279)	0.907 (0.900–0.914)	0.810 (0.794–0.827)	0.840 (0.832–0.849)	0.609 (0.591–0.628)	0.935 (0.929–0.941)	0.695 (0.679–0.710)
Phillips (N=4,720)	0.906 (0.898–0.915)	0.801 (0.781–0.821)	0.853 (0.844–0.862)	0.631 (0.611–0.651)	0.932 (0.925–0.939)	0.706 (0.689–0.722)
GE (N=1,022)	0.913 (0.896–0.931)	0.810 (0.768–0.850)	0.845 (0.825–0.866)	0.598 (0.553–0.645)	0.940 (0.925–0.954)	0.688 (0.651–0.724)
Acuson (N=508)	0.913 (0.888–0.938)	0.743 (0.673–0.813)	0.901 (0.876–0.924)	0.661 (0.592–0.729)	0.931 (0.909–0.952)	0.700 (0.640–0.755)
**Diastolic Dysfunction**						
All data (N=2263)	0.836 (0.821–0.851)	0.736 (0.718–0.755)	0.774 (0.747–0.801)	0.878 (0.864–0.893)	0.570 (0.544–0.596)	0.801 (0.787–0.815)
Phillips (N=1723)	0.824 (0.806–0.843)	0.757 (0.736–0.777)	0.726 (0.695–0.757)	0.868 (0.850–0.885)	0.557 (0.523–0.590)	0.808 (0.794–0.824)
GE (N=394)	0.907 (0.880–0.930)	0.659 (0.612–0.707)	0.953 (0.924–0.979)	0.959 (0.933–0.982)	0.627 (0.572–0.677)	0.781 (0.746–0.816)
**Valve Regurgitation**						
All data (N=4148)	0.904 (0.892–0.915)	0.826 (0.796–0.854)	0.829 (0.818–0.839)	0.395 (0.369–0.421)	0.972 (0.967–0.977)	0.534 (0.509–0.560)
Phillips (N=2721)	0.907 (0.894–0.921)	0.825 (0.792–0.860)	0.837 (0.825–0.849)	0.408 (0.379–0.439)	0.972 (0.967–0.978)	0.546 (0.517–0.576)
GE (N=1069)	0.915 (0.897–0.933)	0.814 (0.770–0.855)	0.864 (0.845–0.885)	0.630 (0.581–0.678)	0.942 (0.928–0.956)	0.710 (0.669–0.749)
Acuson (N=271)	0.920 (0.892–0.948)	0.852 (0.772–0.925)	0.820 (0.778–0.863)	0.541 (0.453–0.630)	0.957 (0.930–0.979)	0.662 (0.583–0.731)

Legend: (DNN) Deep Neural Network, (AUC) Area under the receiver operating characteristic curve, (PPV) Positive Predictive Value, (NPV) Negative Predictive Value, (CI) Confidence Interval. All values are point estimate (95% confidence interval, derived via bootstrap).

## Code availability

The multiview DNN architecture for non-commercial purposes is available at https://github.com/JoshBarrios/omniread-echo. The code that supports this work is copyright of the Regents of the University of California and can be made available for commercial purposes through license.
