# Supplementary material for: Multiview deep learning improves detection of major cardiac conditions from echocardiography
Source: Nat Cardiovasc Res. 2026 Mar 17;5(3):234–45. doi: 10.1038/s44161-026-00786-7 (PMC12995717; doi:10.1038/s44161-026-00786-7)
Supplement: Supplementary file 1 — Discussion and references. [file 44161_2026_786_MOESM1_ESM.pdf]

nature cardiovascular research

Supplementary information https://doi.org/10.1038/s44161-026-00786-7

Multiview deep learning improves detection of major cardiac conditions from echocardiography

In the format provided by the authors and unedited

Extended Data Discussion

For diastolic dysfunction, few prior efforts have used DNNs to analyze raw echo image data, with most instead applying machine learning analysis to echo-derived measurements[1–3]. These efforts differ substantially in being reliant on measurements derived from the raw echo data rather than analyzing the raw echo data itself. Derived measurements require upstream physician analysis or algorithms to first derive these measurements from the raw echo videos which introduces various limitations and therefore is substantially different from our approach. A recent effort by Chen et al. compared several different approaches, including using derived measurements and direct prediction, to grade diastolic dysfunction[4]. For one approach, they first used a DNN to segment and derive measurements from echo videos, then they used the derived metrics from multiple views to identify diastolic dysfunction grades. As an alternate approach, they also examined single-view models that considered specific measurements like left atrial or LV strain to classify diastolic dysfunction. Importantly, using single measurements like left atrial or LV strain to predict diastolic dysfunction provides only a limited approximation of a comprehensive diastolic function assessment. As another approach, they also trained DNNs to predict diastolic dysfunction directly from raw echo data from the single A4c view, reporting AUCs of 0.81-0.88[4]. Their approach of examining non-raw data from multiple echo views is fundamentally different from our multi-view DNN architecture in being specifically tailored to diastolic dysfunction only and not providing a generalizable DNN architecture that can be applied to other echo tasks or imaging modalities. It bears greater similarity to the other prior efforts that used derived measurements to discriminate diastolic dysfunction. Our work shows that for

the diastolic dysfunction task, a DNN that considers multiple views of raw echo data directly can improve discriminative performance over a single view alone.

Here we discuss a few additional study limitations. Our multi-view DNNs were trained on retrospective data acquired from UCSF from 2012-2020 which is limited to a single center. We attempted to mitigate this by performing external validation in MHI. Our model generalized fairly well to MHI external data with only modest performance degradation for some tasks. Several factors may have contributed to the lower multi-view DNN performance in MHI data. MHI used linear measurements to identify abnormal ventricular size compared to UCSF's volumetric measurements, effectively requiring us to use different definitions for abnormal chamber size for MHI echos. Practice patterns at MHI may also differ in the qualitative assessment of valvular regurgitation severity and diastolic function assessment. Likely in part due to these differences, we observed meaningful demographic and disease-prevalence differences between the UCSF and MHI datasets: the MHI dataset had more abnormal LV ejection fraction, fewer higher-grade diastolic dysfunction and more mitral valve regurgitation compared to UCSF. The distribution of echo hardware manufacturers significantly differed between UCSF and MHI, with MHI being 100% General Electric and UCSF being 59% Philips and only 15% General Electric; differences in echo video acquisition between vendors may have contributed to performance degradation. It is also possible that errors made by our UCSF-trained view classifier on MHI external validation data contributed to our observed performance degradation of multi-view DNNs on MHI if incorrect views were used as input. On manual review of the view classifier's predictions on a

subset of the MHI dataset, however, we found error rates comparable to UCSF, suggesting that this was not likely a major contributor to performance differences.

An additional limitation is that color Doppler videos were not classified with respect to which valve was the focus of the doppler window, which likely caused some performance degradation of our valve regurgitation models. There is also the limitation that the UCSF labels used for training were generated during clinical care which may be prone to error compared to dedicated research-generated labels. However, all clinical interpretations were made by level 3 board certified echocardiographers at the UCSF echo lab, and label error would be expected to bias the observed DNN performance towards null. Finally, due to the relatively large nature of our dataset and the computational requirements of the multi-view model, it was not computationally feasible to perform k-fold cross validation across all of our models in a reasonable time frame. However, the MHI external validation results suggest that cross-validation would not likely change our primary findings.

Extended Data Discussion References

1. Chiou, Y. A., Hung, C. L. & Lin, S. F. AI-Assisted Echocardiographic Prescreening of Heart Failure With Preserved Ejection Fraction on the Basis of Intrabeat Dynamics. *JACC Cardiovasc Imaging* 14, 2091–2104 (2021).
2. Tromp, J. *et al.* Automated interpretation of systolic and diastolic function on the echocardiogram: a multicohort study. *Lancet Digit Health* 4, e46–e54 (2022).
3. Pandey, A. *et al.* Deep-Learning Models for the Echocardiographic Assessment of Diastolic Dysfunction. *JACC Cardiovasc Imaging* 14, 1887–1900 (2021).
4. Chen, X. *et al.* Artificial Intelligence–Assisted Left Ventricular Diastolic Function Assessment and Grading: Multiview Versus Single View. *Journal of the American Society of Echocardiography* 36, 1064–1078 (2023).
