# Supplementary material for: Multiview deep learning improves detection of major cardiac conditions from echocardiography
Source: Nat Cardiovasc Res. 2026 Mar 17;5(3):234–45. doi: 10.1038/s44161-026-00786-7 (PMC12995717; doi:10.1038/s44161-026-00786-7)
Supplement: Supplementary file 2 — Reporting Summary [file 44161_2026_786_MOESM2_ESM.pdf]

nature portfolio

Corresponding author(s): Geoffrey H. Tison

Last updated by author(s): Jan 30, 2026

Reporting Summary

Nature Portfolio wishes to improve the reproducibility of the work that we publish. This form provides structure for consistency and transparency in reporting. For further information on Nature Portfolio policies, see our Editorial Policies and the Editorial Policy Checklist.

Statistics

For all statistical analyses, confirm that the following items are present in the figure legend, table legend, main text, or Methods section.

n/a	Confirmed	
☐	☒	The exact sample size (*n*) for each experimental group/condition, given as a discrete number and unit of measurement
☐	☒	A statement on whether measurements were taken from distinct samples or whether the same sample was measured repeatedly
☐	☒	The statistical test(s) used AND whether they are one- or two-sided<br>*Only common tests should be described solely by name; describe more complex techniques in the Methods section.*
☒	☐	A description of all covariates tested
☒	☐	A description of any assumptions or corrections, such as tests of normality and adjustment for multiple comparisons
☐	☒	A full description of the statistical parameters including central tendency (e.g. means) or other basic estimates (e.g. regression coefficient) AND variation (e.g. standard deviation) or associated estimates of uncertainty (e.g. confidence intervals)
☐	☒	For null hypothesis testing, the test statistic (e.g. *F*, *t*, *r*) with confidence intervals, effect sizes, degrees of freedom and *P* value noted<br>*Give P values as exact values whenever suitable.*
☒	☐	For Bayesian analysis, information on the choice of priors and Markov chain Monte Carlo settings
☒	☐	For hierarchical and complex designs, identification of the appropriate level for tests and full reporting of outcomes
☒	☐	Estimates of effect sizes (e.g. Cohen's *d*, Pearson's *r*), indicating how they were calculated

Our web collection on statistics for biologists contains articles on many of the points above.

Software and code

Policy information about availability of computer code

Data collection	Echocardiography videos were acquired using commercial ultrasound machines from Philips and General Electric. Video data were exported in DICOM format and preprocessed using custom Python 3.8.8 scripts for cropping, resizing, and masking, implemented with the PyTorch 1.8.8 library and standard Python imaging and scientific libraries (e.g. OpenCV, NumPy). No commercial software was used for data collection beyond standard clinical equipment.
Data analysis	All data analysis, deep neural network training, and evaluation were performed in Python 3.8.8 using the PyTorch library version 1.8.8. Custom code was developed for training single-view and multi-view video neural networks, including model architecture design and hyperparameter optimization. Statistical and numerical analyses were conducted in Python using pandas 2.3.0, numpy 1.26.4, scikit-learn 1.6.1, statsmodels 0.14.5, and MLstatkit 0.1.7.

For manuscripts utilizing custom algorithms or software that are central to the research but not yet described in published literature, software must be made available to editors and reviewers. We strongly encourage code deposition in a community repository (e.g. GitHub). See the Nature Portfolio guidelines for submitting code & software for further information.

Data

Policy information about availability of data

All manuscripts must include a data availability statement. This statement should provide the following information, where applicable:

- Accession codes, unique identifiers, or web links for publicly available datasets
- A description of any restrictions on data availability
- For clinical datasets or third party data, please ensure that the statement adheres to our policy

The full clinical datasets from UCSF and MHI cannot be publicly shared due to patient privacy constraints. Researchers may request access for collaboration purposes, subject to institutional approvals.

Research involving human participants, their data, or biological material

Policy information about studies with human participants or human data. See also policy information about sex, gender (identity/presentation), and sexual orientation and race, ethnicity and racism.

Reporting on sex and gender	Sex was reported as male or female in the demographic tables and summary statistics. Analyses were stratified by sex to examine model performance differences. No analyses were performed based on gender identity.
Reporting on race, ethnicity, or other socially relevant groupings	Race and ethnicity were reported as demographic characteristics in Table 1, including categories for White, Asian, Black, Latinx, and Other. These variables were extracted from structured clinical records. No analyses were performed to adjust for race or ethnicity, nor were these variables used as covariates in model training or evaluation.
Population characteristics	The study population included adult patients undergoing echocardiography at UCSF between 2012-2020 and at the Montreal Heart Institute in 2022. Mean age ranged from 61-65 years across cohorts. Approximately 50% of participants were male. Demographic information including race, ethnicity, and cardiovascular comorbidities (e.g. hypertension, diabetes, coronary artery disease) is provided in Table 1. All participants had clinical indications for echocardiography. The external validation cohort differed slightly in disease prevalence and echo acquisition characteristics.
Recruitment	Participants were not prospectively recruited. This study used retrospectively collected echocardiography video data from routine clinical care. Potential biases include differences in disease prevalence and practice patterns between institutions, which may impact generalizability. No direct participant consent was required for this retrospective analysis.
Ethics oversight	This study was approved by the Institutional Review Boards of the University of California, San Francisco and the University of Montreal.

Note that full information on the approval of the study protocol must also be provided in the manuscript.

Field-specific reporting

Please select the one below that is the best fit for your research. If you are not sure, read the appropriate sections before making your selection.

☒ Life sciences ☐ Behavioural & social sciences ☐ Ecological, evolutionary & environmental sciences

For a reference copy of the document with all sections, see nature.com/documents/nr-reporting-summary-flat.pdf

Life sciences study design

All studies must disclose on these points even when the disclosure is negative.

Sample size	No formal sample size calculation was performed. Sample sizes were determined by the availability of clinical echocardiogram studies in the UCSF and MHI databases. The large size of the datasets (e.g. ~20,504 patients for LV/RV abnormality) was considered sufficient for model training and robust statistical evaluation, as evidenced by narrow confidence intervals around performance metrics.
Data exclusions	Studies of patients aged <18 were excluded. Studies were excluded if they were missing any of the required echocardiographic views necessary for multi-view or single-view model input. Transesophageal, intracardiac, and stress echocardiography studies were also excluded, as their imaging characteristics differ substantially from standard transthoracic echo. These exclusion criteria were pre-established to ensure consistent data inputs for model training.
Replication	No experimental replication was performed for this retrospective analysis. Reproducibility was tested via external validation on an independent dataset from the Montreal Heart Institute (MHI). Performance was comparable for some tasks but showed modest degradation in others, reflecting differences in imaging protocols and patient populations. No experimental findings were irreproducible within the datasets studied.
Randomization	Data were randomly split by patient into training, development, and test sets in a 70/15/15 ratio to avoid overlap of patient data across datasets. No other randomization procedures were applicable because this was a retrospective analysis without experimental interventions.
Blinding	The analysis was performed retrospectively. No blinding was performed during data analysis because group allocation was not applicable to

Blinding	this study design. Analyses focused on model development and evaluation using labeled data from clinical reports. Investigators did not influence data collection or labeling.

Reporting for specific materials, systems and methods

We require information from authors about some types of materials, experimental systems and methods used in many studies. Here, indicate whether each material, system or method listed is relevant to your study. If you are not sure if a list item applies to your research, read the appropriate section before selecting a response.

Materials & experimental systems

n/a	Involved in the study
☒	☐ Antibodies
☒	☐ Eukaryotic cell lines
☒	☐ Palaeontology and archaeology
☒	☐ Animals and other organisms
☒	☐ Clinical data
☒	☐ Dual use research of concern
☒	☐ Plants

Methods

n/a	Involved in the study
☒	☐ ChIP-seq
☒	☐ Flow cytometry
☒	☐ MRI-based neuroimaging

Plants

Seed stocks	*Report on the source of all seed stocks or other plant material used. If applicable, state the seed stock centre and catalogue number. If plant specimens were collected from the field, describe the collection location, date and sampling procedures.*
Novel plant genotypes	*Describe the methods by which all novel plant genotypes were produced. This includes those generated by transgenic approaches, gene editing, chemical/radiation-based mutagenesis and hybridization. For transgenic lines, describe the transformation method, the number of independent lines analyzed and the generation upon which experiments were performed. For gene-edited lines, describe the editor used, the endogenous sequence targeted for editing, the targeting guide RNA sequence (if applicable) and how the editor was applied.*
Authentication	*Describe any authentication procedures for each seed stock used or novel genotype generated. Describe any experiments used to assess the effect of a mutation and, where applicable, how potential secondary effects (e.g. second site T-DNA insertions, mosiacism, off-target gene editing) were examined.*
